# Topical Apigenin Alleviates Cutaneous Inflammation in Murine Models

**DOI:** 10.1155/2012/912028

**Published:** 2012-11-19

**Authors:** Mao-Qiang Man, Melanie Hupe, Richard Sun, George Man, Theodora M. Mauro, Peter M. Elias

**Affiliations:** ^1^The Center for Skin Physiology Research, Dalian Skin Disease Hospital, Liaoning 116021, China; ^2^Dermatology Service, Veterans Affairs Medical Center and Department of Dermatology, University of California, San Francisco, 4150 Clement Street, San Francisco, CA 94121, USA

## Abstract

Herbal medicines have been used in preventing and treating skin disorders for centuries. It has been demonstrated that systemic administration of chrysanthemum extract exhibits anti-inflammatory properties. However, whether topical applications of apigenin, a constituent of chrysanthemum extract, influence cutaneous inflammation is still unclear. In the present study, we first tested whether topical applications of apigenin alleviate cutaneous inflammation in murine models of acute dermatitis. The murine models of acute allergic contact dermatitis and acute irritant contact dermatitis were established by topical application of oxazolone and phorbol 12-myristate 13-acetate (TPA), respectively. Inflammation was assessed in both dermatitis models by measuring ear thickness. Additionally, the effect of apigenin on stratum corneum function in a murine subacute allergic contact dermatitis model was assessed with an MPA5 physiology monitor. Our results demonstrate that topical applications of apigenin exhibit therapeutic effects in both acute irritant contact dermatitis and allergic contact dermatitis models. Moreover, in comparison with the vehicle treatment, topical apigenin treatment significantly reduced transepidermal water loss, lowered skin surface pH, and increased stratum corneum hydration in a subacute murine allergic contact dermatitis model. Together, these results suggest that topical application of apigenin could provide an alternative regimen for the treatment of dermatitis.

## 1. Introduction

 Increasing evidence demonstrates the beneficial effects of herbal medicines in preventing and treating a variety of disorders, including inflammatory dermatoses. Prior studies have shown that topical applications of herbal extract prevent acute irritant murine contact dermatitis [1] and that oral administration of *Cordyceps sinensis* extract prevents cutaneous damages from *Streptococcus pyogenes *infection in an air pouch murine infection model [2]. Likewise, oral administration of *Hainosankyuto* reduces skin lesion size and increased survival rate in a *Streptococcus pyogenes* infected murine model [3]. Similarly, oral administration of the water extract of *Astragalus membranaceus* inhibits the development of atopic dermatitis-like lesions in a murine model [4]. Moreover, topical herbal extracts protect skin from UV radiation [5–7]. Additionally, *Scutellaria bardata* exhibits a preventive effect on the development of skin cancer [8]. Furthermore, the therapeutic effects of some herbal medicines on cutaneous inflammation have been well studied in both animals and humans. For instance, previous studies demonstrated that either topical or oral applications of herbal extracts inhibited both acute cutaneous inflammation and atopic dermatitis in animal models [9–12]. Study revealed that the majority of atopic dermatitis patients accepted herbal medicines as an alternative treatment approach [13]. Clinically, oral administrations of herbal medicines are effective in treating psoriasis, atopic dermatitis, and glucocorticoid-induced dermatitis [14–17]. 

Chrysanthemum is a common herbal medicine. The anti-inflammatory effects of chrysanthemum have been documented. For example, systemic administration of chrysanthemum extract inhibits both acute and chronic irritant contact dermatitis in murine models [18]. Topical applications of chrysanthemum extract alleviate diaper dermatitis in infants and newborns with erythema venenatum [19, 20]. Improvement of certain cutaneous drug reactions also has been reported with chrysanthemum [21]. Apigenin is an active constituent that is present in large quantities in chrysanthemum extract [22, 23]. It has been shown that apigenin exhibits preventive activity against UVB-induced cyclooxygenase-2 (COX-2) expression in keratinocyte cultures [24, 25]. In a murine model, an apigenin-enriched diet attenuated the development of atopic dermatitis-like lesions [26]. Although one clinical study showed that an apigenin containing cream inhibited cutaneous inflammation [27], the therapeutic effects of topical apigenin on cutaneous inflammation and barrier function have previously not been elucidated yet. In the present study, we evaluated the effects of topical apigenin on both acute and subacute cutaneous inflammation in murine models. 

## 2. Materials and Methods

### 2.1. Materials

Both 6–8-week-old female hairless mice (hr/hr) and C57BL/6J mice were purchased from Charles River Laboratories (Wilmington, MA, USA) and fed mouse diet (Ralston-Purina Co., St Louis, MO, USA) and water *ad libitum*. Apigenin powder was from Sigma Chemical Co. (St Louis, MO, USA). Phorbol 12-myristate 13-acetate (TPA), 4-Ethoxymethylene-2-phenyloxazol-5-one (oxazolone), and ethanol were purchased from Sigma Chemical Co. (St Louis, MO, USA).

### 2.2. Experimental Protocols and Functional Studies

All animal procedures were approved by the Animal Studies Subcommittee (IACUC) of the San Francisco Veterans Administration Medical Center and performed in accordance with their guidelines. For anti-inflammatory studies in irritant contact dermatitis model, ear inflammation on C57BL/6J mice was induced by a topical application of 15 *μ*L of 0.03% TPA to both the inner and outer surfaces of both ears [28, 29]. 20 *μ*L of 0.1% (about 0.67 mg/kg body weight) apigenin in ethanol was applied to both surfaces of the right and 20 *μ*L of ethanol alone was applied to both surfaces of the left ear at 45 min and 2 hours following TPA treatment. Additionally, the oxazolone-induced ear inflammation model (allergic contact dermatitis model) was also used to assess the anti-inflammatory effects of apigenin. C57BL/6J mice were sensitized by topical application of 3% oxazolone to the back once daily for two days. One week later, 15 *μ*L of 0.5% oxazolone was applied to both the inner and outer surfaces of both ears. 20 *μ*L of 0.1% apigenin or ethanol alone was applied to both surfaces of the right or left ear at 45 min and 2 hours following oxazolone treatment. Ear thickness was measured with a digital caliper (Mitutoyo, Tokyo, Japan) before and 20 hours after the challenge with oxazolone or TPA application. Under anesthesia (4% chlorohydrate, IP injection) ear samples were taken with surgical scissors for hematoxylin and eosin staining (H&E) staining of 5 **μ**m paraffin-enabled sections [30].

 For the subacute dermatitis model, 6–8-week-old female hairless mice (hr/hr) with body weight of 28–30 g were sensitized by topical application of 3% oxazolone to the back once daily for two days. One week later, 60 *μ*L of 0.01% oxazolone was applied to both flanks of mice once every other day for 4 applications. One group of oxazolone-treated mice was topically treated with 60 *μ*L of 0.1% (about 2 mg/kg body weight) apigenin twice daily for 7 days. The other group of oxazolone-treated mice was topically treated with ethanol alone and served as the control. In the case that oxazolone and apigenin or ethanol were applied on the same day, apigenin and ethanol were applied one hour after oxazolone application. On the 8th day, 18 hours after the last apigenin or ethanol application, basal transepidermal water loss (TEWL), stratum corneum hydration, and skin surface pH were measured using their respective probes connected to MPA5 (C&K, Cologne, Germany) as described earlier [31, 32].

### 2.3. Statistics

Data are expressed as the mean ± SEM. GraphPad Prism 4 software (San Diego, CA, USA) was used for all statistical analyses. Unpaired two-tailed Student's  *t*-test with Welch's correction was used to determine the statistical significances when two groups were compared. One-Way ANOVA with Tukey correction was used when three or more groups were compared.

## 3. Results

### 3.1. Topical Apigenin Inhibits Acute Cutaneous Inflammation in Murine Contact Dermatitis Models

**Figure 1:**
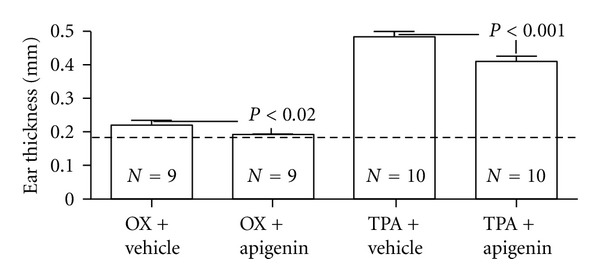
Topical apigenin reduces ear thickness in acute murine dermatitis models. Acute irritant contact dermatitis and allergic contact dermatitis models were established as described in Materials and Methods. 20 *μ*L of 0.1% apigenin in ethanol or ethanol alone was applied to both surfaces of the right or left ear, respectively, 45 min and 2 hours following TPA or oxazolone challenge. Ear thickness was measured with a digital caliper (Mitutoyo, Tokyo, Japan) before and 20 hours after challenge with oxazolone or TPA application. The dotted line represented the normal ear thickness. Numbers and significances are indicated in the figures.

**Figure 2:**
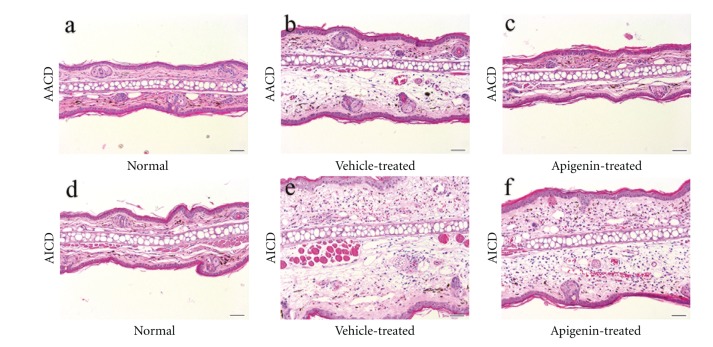
Topical applications of apigenin reduce edema in acute murine dermatitis models. Acute irritant contact dermatitis and allergic contact dermatitis models were established as described in Materials and Methods Ear samples for H&E staining were taken immediately after measurements of ear thickness. (a) and (d) are normal ears. (b) and (c) are acute allergic contact dermatitis (AACD) treated with vehicle and apigenin, respectively. (e) and (f) are acute irritant contact dermatitis (AICD) treated with vehicle and apigenin, respectively. A remarkable reduction in ear thickness and edema were evident in apigenin-treated ear. The magnifications for all images are the same. Scale bar = 50 *μ*m.

 We first assessed whether topical applications of apigenin inhibit acute irritant contact dermatitis (AICD) and acute allergic contact dermatitis (AACD) in murine dermatitis models. As seen in Figure 1, ear thickness increased following either TPA or oxazolone treatment. Topical applications of apigenin almost completely normalized ear thickness in a murine AACD model (0.186 ± 0.003 for normal; 0.189 ± 0.003 for oxazolone + apigenin). Similarly, topical apigenin significantly reduced ear thickness in murine AICD model (0.485 ± 0.013 for TPA + vehicle; 0.409 ± 0.016 for TPA + apigenin, *P* < 0.001) (Figure 1). The anti-inflammatory effects of apigenin on acute cutaneous inflammation were further confirmed by H&E staining (Figure 2). These results demonstrate that topical apigenin inhibits acute cutaneous inflammation in murine dermatitis models.

### 3.2. Topical Apigenin Lowers Transepidermal Water Loss in a Subacute Murine Allergic Contact Dermatitis Model

**Figure 3:**
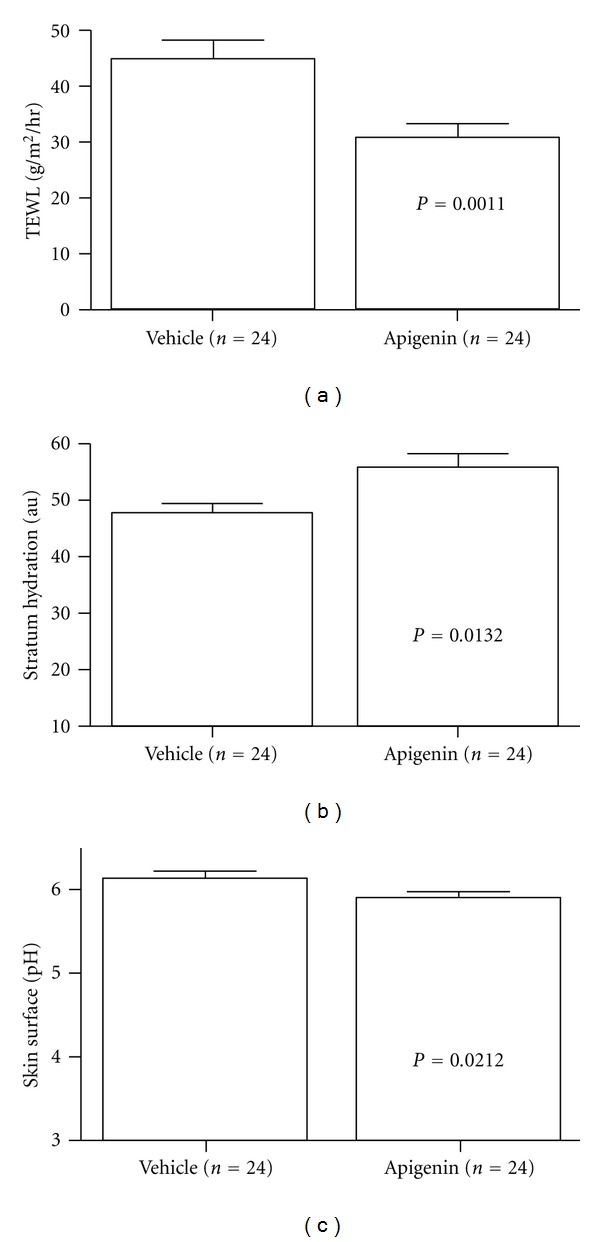
Topical apigenin improves stratum corneum function in murine model of subacute allergic contact dermatitis. Subacute dermatitis model was established as described in Materials and Methods. On the 8th day, basal transepidermal water loss, skin surface pH, and stratum corneum (SC) hydration were assessed with an MPA5 (CK electronic GmbH, Cologne, Germany) connected to TM 300, pH905, and Corneometer 825. Two readings were taken from each mouse for basal TEWL, hydration, as well as pH. (a) indicates a reduction in transepidermal water loss following apigenin treatment; (b) shows apigenin induced an increase in stratum corneum hydration; (c) exhibits a lower skin surface pH after apigenin treatment. Numbers and significances are indicated in the figures.

 Previous studies have demonstrated that apigenin attenuates the development of atopic dermatitis-like lesions [26] and that transepidermal water loss positively correlates with the severity of subacute and chronic dermatitis [33, 34]. We next determined whether topical apigenin influences transepidermal water loss in subacute cutaneous inflammation in a murine model. As shown in Figure 3(a), repeated topical oxazolone treatment markedly increased transepidermal water loss as compared with vehicle-treated control (*P* < 0.001). Topical apigenin treatment dramatically prevented the increase in transepidermal water loss induced by oxazolone treatment (*P* < 0.001 versus oxazolone + vehicle treatment). This result indicates that topical apigenin improves epidermal permeability barrier function in murine subacute dermatitis model.

 It has been shown that subacute and chronic dermatitis are characterized by lower stratum corneum hydration and higher skin surface pH [30, 35]. Therefore, stratum corneum hydration and skin surface pH were also evaluated following vehicle and apigenin treatment in oxazolone-treated mice. As reported previously [30], repeated oxazolone applications significantly decreased stratum corneum hydration (Figure 3(b)). Although the apigenin treatment did not normalize stratum corneum hydration, a notably higher stratum corneum hydration was observed following apigenin treatment (Figure 3(b), *P* < 0.05 for oxazolone + vehicle versus oxazolone + apigenin). In agreement with prior findings [30], repeated oxazolone treatment caused a significant increase in skin surface pH (Figure 3(c), *P* < 0.001 for normal versus oxazolone + vehicle). In comparison with the vehicle treatment, a substantially lower skin surface pH was apparent in apigenin-treated mice (Figure 3(c), *P* < 0.05 for oxazolone + vehicle versus oxazolone + apigenin; unpaired Student's  *t*-test). These results suggest that topical apigenin partially inhibits the changes of stratum corneum hydration and skin surface pH induced by repeated oxazolone applications.

Together, these results demonstrate that topical apigenin attenuates the changes of stratum corneum function induced by repeated oxazolone applications. 

## 4. Discussion

 It has been shown that topical applications of herbal extracts inhibit cutaneous inflammation and improve both the epidermal permeability barrier and the antimicrobial barrier function [1, 9, 32]. However, the active constituents of herbal medicines have not yet been well defined. Recently, an active ingredient, hesperidin, in orange peel has been shown to improve the epidermal permeability barrier function [31]. In the present study, we first demonstrated that topical applications of apigenin, an extract from chrysanthemum, inhibit both acute irritant and acute allergic dermatitis in murine models. Although the exact mechanisms by which apigenin inhibits acute cutaneous inflammation are not clear, several potential mechanisms could be involved. It is well known that matrix metalloproteinase-1 is involved in cutaneous inflammation [36, 37]. It has been reported that apigenin inhibits matrix metalloproteinase-1 expression induced by 12-O-tetradecanoylphorbol 13-acetate in dermal fibroblasts [38]. Secondly, in addition to inhibiting TNF-alpha gene expression induced by lipopolysaccharide [39], apigenin also inhibits TNF-alpha secretion *in vitro* [40]. Similarly, oral administrations of apigenin reduce the high serum TNF-alpha levels induced by romurtide in mice [41]. Thirdly, apigenin inhibits the expression of inflammation-related molecules, such as intercellular adhesion molecule-1, vascular cell adhesion molecule-1, and E-selectin, induced by TNF-alpha and IL-1alpha [42, 43]. Studies suggest that inhibition of inflammation by apigenin is via nuclear factor (NF)-*κ*B and MAPKs pathways [44, 45]. All these anti-inflammatory effects induced by apigenin are likely attributed to its antioxidant properties. Apigenin is a well-known antioxidant [46, 47]. Studies have demonstrated that antioxidants such as quercetin (Que) and cromolyn inhibit release of inflammatory mediators including histamine, leukotrienes, IL-6, IL-8, and TNF release from mast cells *in vitro* [48]. *In vivo* studies reveal that vitamin E, an antioxidant, improves atopic dermatitis-like inflammation [49]. Therefore, the anti-inflammatory effect of apigenin could be due to its antioxidant effects. 

 Previous studies revealed that stratum corneum function, especially transepidermal water loss, positively correlates with the severity of atopic dermatitis [50, 51]. And inhibition of cutaneous inflammation could decrease transepidermal water loss [52, 53]. In the present study, the effect of apigenin on subacute dermatitis was evaluated by assessing transepidermal water loss, a parameter of epidermal permeability barrier function. A significantly lower level of transepidermal water loss was observed in apigenin-treated mice. This improvement of transepidermal water loss could be attributable to both the anti-inflammatory and the antioxidant properties of apigenin. Thus, apigenin-induced improvement of transepidermal water loss in the murine subacute dermatitis model is at least partially due to the inhibition of cutaneous inflammation. In addition, apigenin exhibits antioxidant properties [54, 55]. It has been shown that both systemic and topical administration of antioxidants lower transepidermal water loss [56–59]. Hence, the apigenin-induced improvement in epidermal permeability barrier homeostasis could result from its antioxidant properties.

 It is worth noting that apigenin is a relatively safe agent. Singh et al. reported that 50 mg/kg body weight of apigenin caused no changes in serum biomarkers (alanine amino transferase, aspartate amino transferase, and alkaline phosphatase) of hepatotoxicity at 48 hours after intraperitoneal injection in Swiss mice [60]. Likewise, no sign of illness was observed in mice after 10 days of single intraperitoneal injection of 40 mg/kg body weight of apigenin [61]. However, apigenin at dosage of 10 mg/kg body weight significantly inhibits cytokines such as TNF, IL-1 and IL-6 expression *in vivo* [61]. In the present, our results indicated that a lower dose (1.23 mg/kg body weight) apigenin could improve acute dermatoses and topical applications of apigenin at dosage of 4 mg/kg body weight per day for 7 days could relieve subacute dermatitis. Taken together, these results strongly suggest that apigenin is a safe and effective anti-inflammatory agent, especially for topical use. 

## 5. Conclusions

 The present study demonstrates that topical apigenin inhibits acute inflammation and subacute dermatitis as indicated by improved epidermal permeability barrier function. Therefore, apigenin could be useful in treating acute and subacute dermatitis.
